# Long non‐coding RNA SNHG20 promotes the tumorigenesis of oral squamous cell carcinoma via targeting miR‐197/LIN28 axis

**DOI:** 10.1111/jcmm.13987

**Published:** 2018-11-05

**Authors:** Jie Wu, Wei Zhao, Zhonghou Wang, Xu Xiang, Shengchi Zhang, Lina Liu

**Affiliations:** ^1^ Department of Orthodontics The School and Hospital of Stomatology Tianjin Medical University Tianjin China; ^2^ Department of Emergency & Department of General The School and Hospital of Stomatology Tianjin Medical University Tianjin China; ^3^ The Department of Stomatology of First Affiliated Hospital of BaoTou Medical College Inner Mongolia University of Science and Technology BaoTou China; ^4^ The College of Stomatology of BaoTou Medical College Inner Mongolia University of Science and Technology BaoTou China; ^5^ Department of Maxillofacial Surgery Tianjin Stomatological Hospital Hospital of Stomatology Nankai University Tianjin China; ^6^ Department of Otorhinolaryngology Head and Neck Surgery Tianjin First Central Hospital Tianjin China; ^7^ Department of Prosthodontics Tianjin Stomatological Hospital Hospital of Stomatology Nankai University Tianjin China

**Keywords:** LIN28, miR‐197, oral squamous cell carcinoma, SNHG20, stemness

## Abstract

Long non‐coding RNA (lncRNA) has been verified to participate in the tumour regulation, including oral squamous cell carcinoma (OSCC). Nevertheless, the role of lncRNA SNHG20 on OSCC still remains elusive. Here, we investigate the physiopathologic functions of lncRNA SNHG20 in OSCC tumorigenesis and explore its potential mechanism. LncRNA SNHG20 was up‐regulated in OSCC tissue compared with adjacent non‐tumour tissue. Meanwhile, SNHG20 was overexpressed in cancer stem‐like cells. In vitro and in vivo, loss‐of‐function experiments showed that lncRNA SNHG20 knockdown inhibited proliferative ability, mammosphere‐forming ability, ALDH1 expression, stem factors (LIN28, Nanog, Oct4, SOX2) and tumour growth. Bioinformatics and luciferase reporter assay revealed that miR‐197 targeted the 3′‐untranslated regions of SNHG20 and LIN28 by complementary binding. Validation experiments confirmed the associated functions of SNHG20/miR‐197/LIN28 axis on OSCC proliferation and stemness. In summary, our results reveal the important function of SNHG20/miR‐197/LIN28 axis in the oncogenesis and stemness of OSCC, suggesting the vital role of SNHG20 in OSCC tumorigenesis.

## 1 INTRODUCTION

Oral squamous cell carcinoma (OSCC) is an epithelial neoplasm, acting as one of the most aggressive head and neck cancers in human.^1^, ^2^ At histopathological level, OSCC is characterized squamous differentiation, nuclear pleomorphisms, invasive growth and metastasis.^3^ Numerous pathogenic factors accelerate the tumorigenesis of OSCC, including smoking, alcohol abuse, human papillomavirus infection.^4^ The overall 5‐year survival rate of OSCC patients is still pessimistic. Therefore, it is eagerly to develop effective therapeutic targets for the clinical treatment.

Long non‐coding RNAs (lncRNAs) are a type of non‐coding RNAs (ncRNAs) of 200 nucleotides, playing a vital role in carcinogenesis.^5^ Increasing evidences have revealed that lncRNAs participate in the proliferation, metastasis, apoptosis, angiogenesis and drug resistance.^6^, ^7^ For example, lncRNA LINC00668 is up‐regulated in OSCC tissues and cells, which is negatively correlated with miR‐297 expression in OSCC tissues, promoting OSCC activity through VEGFA signalling.^8^ Moreover, lncRNAs might serve as a biomarker in cancer. For instance, linc‐RoR is found to be overexpressed in undifferentiated tumours and presents closely association with OSCCs tumour recurrence and poor prognosis.^9^

In present research, our team investigate the role of lncRNA SNHG20 on the OSCC tumorigenesis. The overexpression of SNHG20 accelerates the oncogenesis of OSCC through competing endogenous RNA (ceRNA) regulation. Our results conclude that SNHG20/miR‐197/LIN28 axis promotes the tumorigenesis and stemness of OSCC, suggesting the vital role of SNHG20 in OSCC.

## 2 MATERIALS AND METHODS

### 2.1 Patients and tissue specimens

A total of 12 pairs of OSCC patients and matched adjacent non‐tumour tissue were enrolled into this research, who received surgical treatment in the Tianjin Stomatological Hospital between October 2016 and August 2017. The tumour samples were examined by two independent pathologists. The tissue samples were rapidly frozen at −80°C for using. This study was approved by the ethics boards of the Hospitals, and all patients signed informed consent for participation.

### 2.2 Cell lines and culture

OSCC cell lines (SCC9, SCC15, SCC25, Ca9‐22, HSU3, TSCCA, Fadu) and the primary normal human oral keratinocytes (NHOK) cell lines were purchased from the Institute of Biochemistry and Cell Biology of the Chinese Academy of Sciences (Shanghai, China), and routinely cultured according to the recommendation and previously described.^10^ Cells were cultured in RPMI‐1640 medium (Gibco, Waltham, MA, USA) supplemented with 10% FBS (Gibco), 100 U/mL penicillin and 100 μg/mL streptomycin. All cells were cultured in a 5% CO_2_ humidified atmosphere at 37°C. To propagate the CSC‐like fraction of the tumour cells, SCC15 and TSCCA cells were trypsinized and cultured in serum‐free stem cell medium containing Dulbecco's modified Eagle's medium (DMEM) supplemented with 20 ng/mL basic fibroblast growth factor (bFGF), 20 ng/mL epidermal growth factor (EGF) (both from PeproTech, Rocky Hill, NJ, USA), 2% B‐27 supplement, 1 mM L‐glutamine and 1% P/S (all from Invitrogen, Carlsbad, CA, USA). CSC‐SCC15 and CSC‐TSCCA cells were washed in PBS and stained with the anti‐CD44‐FITC monoclonal antibody (BD Biosciences, San Jose, CA, USA). After 30 minutes of incubation in the dark in room temperature (RT) samples were analysed with the Accuri™ C6 personal flow cytometer (BD, Oxford, UK), and the data were assessed using the FlowJo software program (Tree Star, Ashland, OR, USA).

### 2.3 Transfection

All these small interfering RNAs (siRNAs), including si‐SNHG20 and negative controls, and miR‐197 inhibitor were specially synthesized by GenePharma company (Shanghai, China). The transfection was performed using Lipofectamine 2000 (Thermo Fisher Scientific, Inc., Rockford, IL, USA) according to the manufacturer's instructions. Sequences were presented as following: si‐SNHG20‐1, 5′‐GAAUCGAUAGGUCGAGGGGTT‐3′, si‐SNHG20‐2, 5′‐GACAGGCCAUUAGGCCACGCCTT‐3′, si‐SNHG20‐3, 5′‐GAAUUAGGAAGCAUUAGGGGTT‐3′.

### 2.4 Quantitative real‐time polymerase chain reaction

Total RNA was extracted from tissue samples and cell groups using TRIzol reagent (Invitrogen) according to the manufacturer's instructions. The RNA quality and concentration were detected using NanoDrop 1000 Spectrophotometer (Thermo Fisher Scientific, Inc.). Complementary DNA (cDNA) was reversely transcribed from RNA (1 μg) using M‐MLV reverse transcriptase (Invitrogen). The real‐time PCR was performed by Power SYBR Green PCR Master Mix (Applied Biosystems, Foster, CA, USA). Primers used in the section were as following: SNHG20, forward, 5′‐ATGGCTATAAATAGATACACGC‐3′, reverse, 5′‐GGTACAAACAGGGAGGGA‐3′; miR‐197, forward, 5′‐CGGTAGTCTGATACTGTAA‐3′, reverse, 5′‐GTGCTCCGAAGGGGGT‐3′; glyceraldehyde 3‐phosphate dehydrogenase (GAPDH), forward, 5′‐GCACCGTCAAGGCTGAGAAC‐3′, reverse, 5′‐TGGTGAAGACGCCAGTGGA‐3′. GAPDH acted as the endogenous controls. Each expression level as the threshold cycle (Ct) was calculated with the 2^−∆∆Ct^ method and every data was performed in triplicate.

### 2.5 Sphere‐formation assay

Sphere‐formation assay was performed as previously described.^11^ Briefly, OSCC was plated in six‐well plates at density of 5 × 10^4^/well. OSCC were resuspended in 2 mL serum‐free DMEM supplemented with 20 mg/L EGF, 20 mg/L hFGF, 4 U/L insulin and 100 U/mL penicillin/streptomycin. The number of spheroids was counted under a stereomicroscope (Olympus, Tokyo, Japan).

### 2.6 Cell counting kit‐8 assay

The proliferation ability of OSCC cells was measured using cell counting kit‐8 (CCK‐8) (Dojindo Laboratories, Kumamoto, Japan) according to the manufacturer's instructions. Briefly, cells were cultured in 96‐well plates at a density of 1000 cells/well. The absorbance was measured at 450 nm to determine the cell viability every 24 hours. The experiments were independently repeated three times.

### 2.7 Western blot analysis

The total proteins were extracted from cells using radioimmunoprecipitation assay lysis buffer (Pierce, Rockford, IL, USA). The cell lysates were prepared and equivalent amount of protein was separated by SDS‐PAGE for 2 hours. Then, the protein was transfected to polyvinylidene fluoride membranes for 2 hours. After being incubated with primary antibodies at 4°C overnight, the membranes were washed using 1% TBST for 30 minutes. All primary antibodies were provided by Cell Signaling Technology, Inc., Danvers, MA, USA (1:1000 dilution), including targeting LIN28, Nanog, SOX2, Oct4 and GAPDH. Then, they were incubated with secondary antibodies (1:5000) for 2 hours and washed by 1% TBST again. Lastly, the blots were detected by chemiluminescence system (Amersham Biosciences, Piscataway, NJ, USA).

### 2.8 Luciferase reporter assay

For luciferase assay, SCC15 cells were co‐transfected with miR‐197 or pcDNA3.1‐luc vector containing SNHG20 and LIN28 wild‐type or mutant 3′‐untranslated regions (UTR) using lipofectamine 2000 according to the manufacturer's protocol. Cells were seeded in 24‐well plates at 60‐70% confluence for 24 hours. After 48 hours, cells were collected and examined for β‐galactosidase and luciferase activities using the Dual Luciferase Reporter Assay System (Promega, Madison, WI, USA) according to the manufacturer's instructions.

### 2.9 Xenograft mice in vivo assay

CSC‐SCC15 cells (1.0 × 10^6^) stably expressing sh‐NC or sh‐SNHG20 were subcutaneously injected into the frank of nude mice (10 mice, 4 week‐old). Mice were sacrifice after 3 weeks and tumours weight were measured and volumes were calculated using the formula length × width^2^/2. All animal experiments were performed in accordance with the Tianjin Stomatological Hospital Animal Care and Use guidelines.

### 2.10 Statistical analysis

Data were presented as mean ± SD. All results were analysed with GraphPad Prism 6 (GraphPad Software, La Jolla, CA, USA) using Student's *t* tests or one‐way ANOVA. Differences were considered to be a statistical significance at a *P*‐value of <0.05.

## 3 RESULTS

### 3.1 LncRNA SNHG20 was up‐regulated in OSCC tissue samples and stem‐like cells

**Figure 1:**
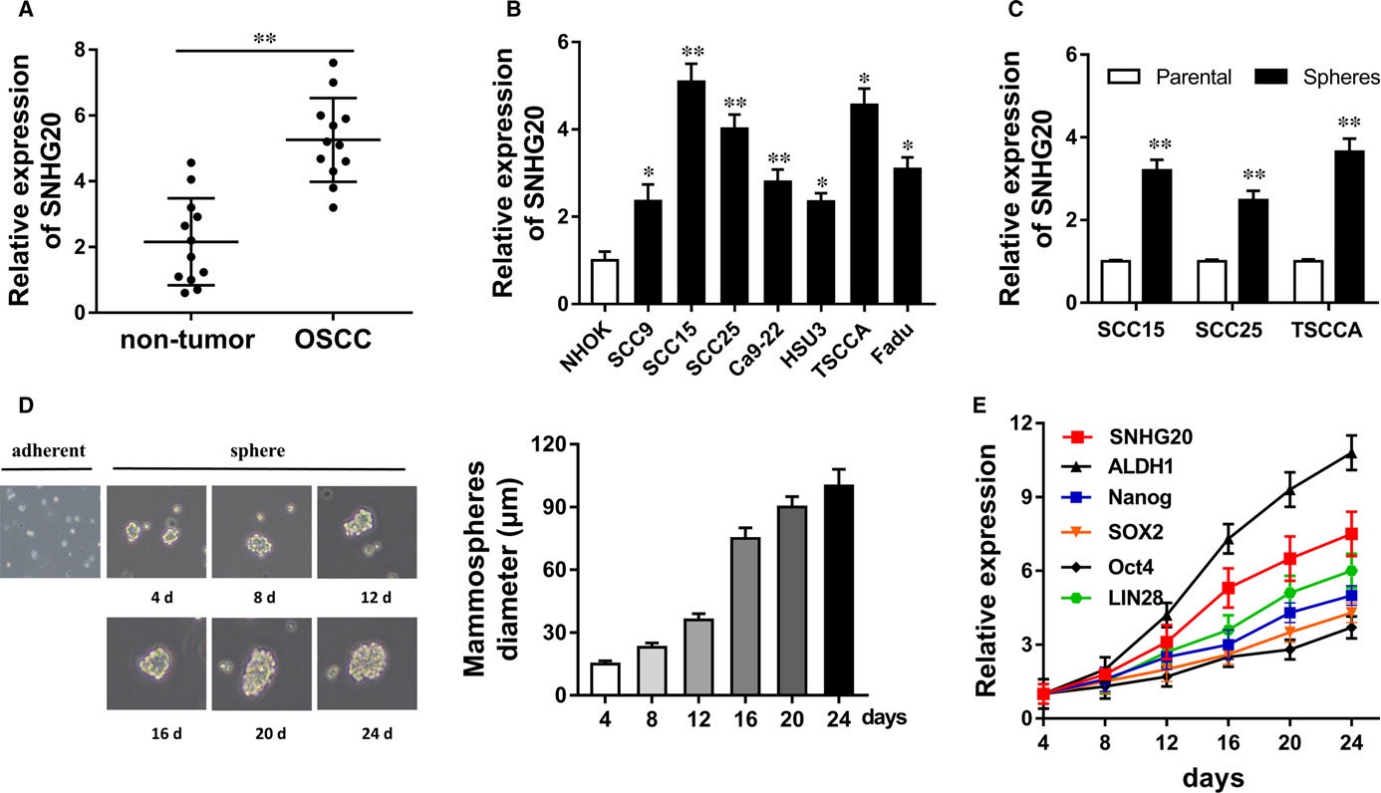
LncRNA SNHG20 was up‐regulated in OSCC tissue samples and stem‐like cells. A, RT‐PCR showed the SNHG20 expression in 12 pairs of OSCC tissue compared with adjacent non‐tumour samples. B, RT‐PCR showed the SNHG20 expression in OSCC cell lines (SCC9, SCC15, SCC25, Ca9‐22, HSU3, TSCCA, Fadu) and the primary normal human oral keratinocytes (NHOK). C, SNHG20 expression in SCC15, SCC25 and TSCCA sphere cells compared with parental cells. D, Mammosphere‐forming assay showed the diameter of OSCC spheres at different culture days. E, RT‐PCR showed the RNA expression levels of SNHG20, ALDH1, Nanog, Oct4 and SOX2. Data are presented as the mean ± SD. *P < 0.05, **P < 0.01 compared to control group

In primary experiments, we measured the expression level of lncRNA SNHG20 in OSCC cells using RT‐PCR. Results showed that SNHG20 expression was up‐regulated in 12 pairs of OSCC tissue compared with adjacent non‐tumour samples (Figure 1A). Meanwhile, RT‐PCR showed that SNHG20 expression was up‐regulated in OSCC cell lines (SCC9, SCC15, SCC25, Ca9‐22, HSU3, TSCCA, Fadu) compared with the primary NHOK (Figure 1B). Moreover, RT‐PCR showed that SNHG20 expression was up‐regulated in OSCC sphere cells compared with parental cells (Figure 1C). Mammosphere‐forming assay showed that the diameter of OSCC spheres was increased with the culture days (Figure 1D). RT‐PCR showed that the RNA expression levels of SNHG20, ALDH1, Nanog, Oct4 and SOX2 were increased following the culture days (Figure 1E). Therefore, results conclude that SNHG20 expression was up‐regulated in OSCC tissue samples and stem‐like cells, suggesting the potential role of SNHG20 on OSCC stemness.

### 3.2 LncRNA SNHG20 knockdown suppressed the CSC properties and tumorigenesis of OSCC in vitro and in vivo

**Figure 2:**
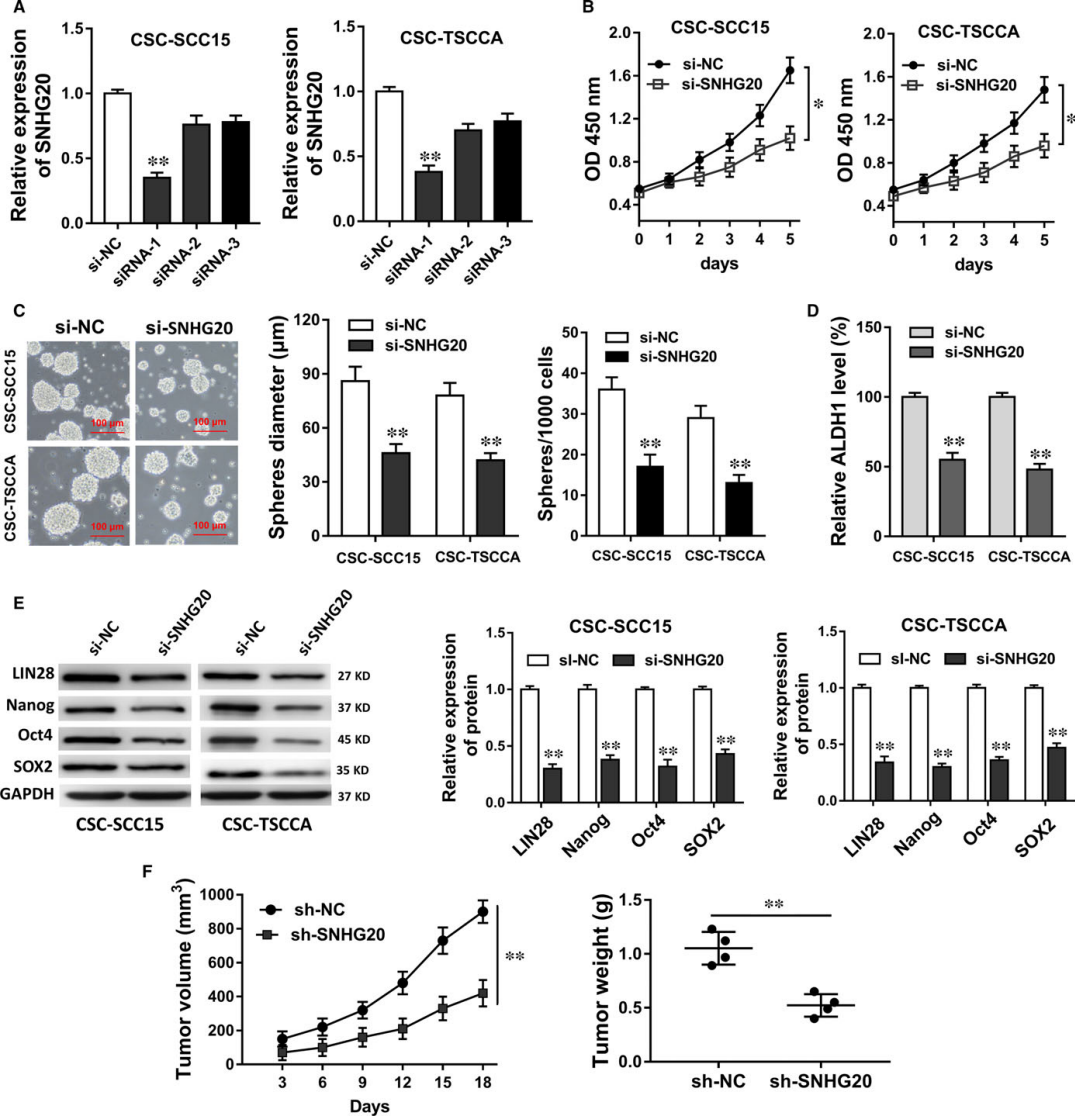
LncRNA SNHG20 knockdown suppressed the CSC properties and tumorigenesis of OSCC in vitro and in vivo. A, RT‐PCR showed the lncRNA SNHG20 expression levels in OSCC stem cells (CSC‐SCC15, CSC‐TSCCA) transfected with siRNAs targeting SNHG20. B, CCK‐8 assay showed the proliferation of OSCC stem cells. C, Sphere formation assays showed the mammosphere‐forming ability (sphere diameter, sphere number). D, RT‐PCR showed the ALDH1 mRNA levels. E, Western blot assay showed the expression levels of stem factors (LIN28, Nanog, Oct4, SOX2). F, Xenograft mice in vivo assay showed the tumour growth of CSC‐SCC15 cells, including tumour volume and weight. Data are presented as the mean ± SD. *P < 0.05, **P < 0.01 compared to control group

To investigate the role of SNHG20 on OSCC stemness and tumorigenesis, loss‐of‐function experiments were performed. LncRNA SNHG20 expression levels were significantly down‐regulated in OSCC stem cells (CSC‐SCC15, CSC‐TSCCA) transfected with siRNAs targeting SNHG20 (Figure 2A). CCK‐8 assay showed that SNHG20 knockdown inhibited the proliferative ability of OSCC cells compared with control group (Figure 2B). Sphere formation assays showed that SNHG20 knockdown decreased the mammosphere‐forming ability of OSCC‐CSC cells (Figure 2C). Besides, RT‐PCR showed that SNHG20 knockdown decreased the ALDH1 mRNA (Figure 2D). Western blot assay showed that SNHG20 knockdown decreased the expression levels of stem factors (LIN28, Nanog, Oct4, SOX2) (Figure 2E). In xenograft mice in vivo assay, SNHG20 knockdown suppressed the tumour growth of OSCC‐CSC (CSC‐SCC15), including tumour volume and weight (Figure 2F). Taken together, results revealed that SNHG20 knockdown suppressed the CSC properties and tumorigenesis of OSCC in vitro and in vivo.

### 3.3 SNHG20 promoted LIN28 through sponging miR‐197

**Figure 3:**
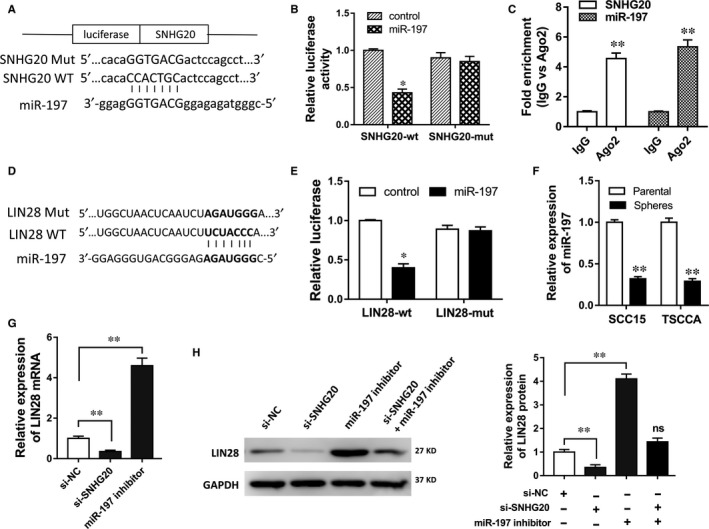
SNHG20 promoted LIN28 through sponging miR‐197. A, Schematic diagram of the interaction within SNHG20 wild‐type (WT), mutant type (Mut) and miR‐197. B, Luciferase reporter assay showed the luciferase activity of SNHG20 WT/Mut and miR‐197. C, Ago2 immunoprecipitate assay illustrated the immunoprecipitate of miR‐197 and SNHG20 in the same RNA‐induced silencing complex. D, Schematic diagram of the interaction within LIN28 WT/Mut and miR‐197. E, Luciferase reporter assay showed the luciferase activity of LIN28 WT/Mut and miR‐197. F, RT‐PCR showed the miR‐197 expression in OSCC sphere cells (SCC15, TSCCA) compared with parental cells. G, RT‐PCR showed the LIN28 mRNA expression in SCC15 cells transfected with si‐SNHG20 or miR‐197 inhibitor. H, Western blot assay showed the LIN28 protein expression in SCC15 cells transfected with si‐SNHG20 and/or miR‐197 inhibitor. Data are presented as the mean ± SD. *P < 0.05, **P < 0.01 compared to control group. ns presented the no significance

Previous experiments verified that SNHG20 knockdown inhibited the stem factors expression; therefore, we performed the bioinformatics programmes and luciferase reporter assay to explore the underlying mechanism. Bioinformatics programmes showed that miR‐197 shared 7‐sites of complementary binding with SNHG20 3′‐UTR (Figure 3A). Then, luciferase reporter assay validated the molecular targeting within SNHG20 and miR‐197 (Figure 3B). Moreover, RIP assay was performed, showing that miR‐197 and SNHG20 were markedly enriched in Ago2 immunoprecipitate of silencing complex comparing with IgG‐pellet (Figure 3C). Meanwhile, bioinformatics tools also predicted that miR‐197 targeted LIN28 mRNA 3′‐UTR (Figure 3D). Similarly, luciferase reporter assay validated the interaction within miR‐197 and LIN28 mRNA (Figure 3E). Moreover, RT‐PCR showed that miR‐197 expression was down‐regulated in OSCC sphere cells (SCC15, TSCCA) compared with parental cells (Figure 3F). RT‐PCR showed that LIN28 mRNA expression was decreased in SCC15 cells transfected with si‐SNHG20, and increased in SCC15 cells transfected with miR‐197 inhibitor (Figure 3G). Similarly, in SCC15 cells, western blot assay showed that LIN28 protein expression was decreased when transfected with si‐SNHG20, while that was increased when transfected with miR‐197 inhibitor (Figure 3H). Moreover, the co‐transfection of si‐SNHG20 and miR‐197 inhibitor reversed the expression of LIN28 protein. Taken together, results concluded that SNHG20 promoted LIN28 protein expression through sponging miR‐197.

### 3.4 SNHG20/miR‐197/LIN28 axis regulated OSCC proliferation and stemness

**Figure 4:**
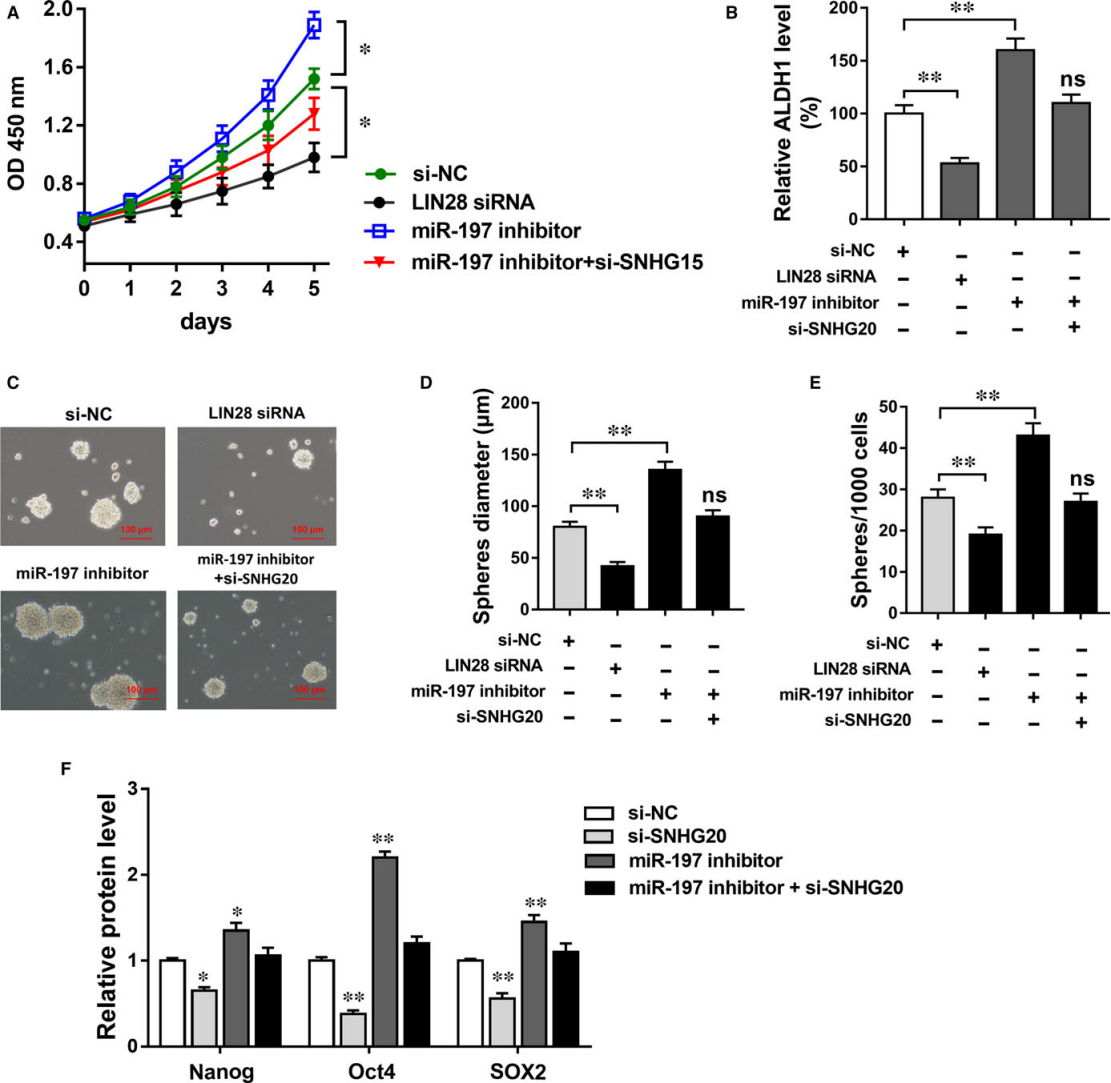
SNHG20/miR‐197/LIN28 axis regulated OSCC proliferation and stemness. A, CCK‐8 assay showed the proliferation of CSC‐SCC15 cells transfected with si‐SNHG20, miR‐197 inhibitor and LIN28 siRNA. B, RT‐PCR showed the ALDH1 expression. C‐E, Sphere formation assays showed the mammosphere‐forming ability (sphere diameter, sphere number). F, Western blot analysis showed the stem factors (Nanog, Oct4, SOX2) expression levels in SCC15 cells co‐transfected with LIN28 siRNA, si‐SNHG20 and miR‐197. Data are presented as the mean ± SD. *P < 0.05, **P < 0.01 compared to control group. ns presented the no significance

Our existing results had indicated that SNHG20 promoted LIN28 through sponging miR‐197. In further experiments, we performed validation experiments to confirm the associated function in CSC‐SCC15 cells. CCK‐8 assay showed that LIN28 siRNA reduced the proliferation ability and miR‐197 inhibitor facilitated the proliferation, while the co‐transfection of si‐SNHG20 and miR‐197 inhibitor rescued the proliferation (Figure 4A). RT‐PCR showed that LIN28 siRNA decreased the ALDH1 expression and miR‐197 inhibitor increased the ALDH1 expression (Figure 4B). Sphere formation assays showed that LIN28 siRNA decreased the mammosphere‐forming ability (sphere diameter, sphere number) of CSC‐SCC15 cells and miR‐197 inhibitor promoted the sphere formation, while the co‐transfection of si‐SNHG20 and miR‐197 inhibitor rescued the sphere formation (Figure 4C‐E). Western blot analysis showed that LIN28 siRNA/miR‐197 inhibitor decreased/increased stem factors (Nanog, Oct4, SOX2) expression, and the co‐transfection of si‐SNHG20 and miR‐197 inhibitor stabilized the stem factors expression levels compared with control group (Figure 4F). Overall, results indicated that SNHG20/miR‐197/LIN28 axis collectively regulated the proliferation and stemness of OSCC, indicating the role of SNHG20, miR‐197 and LIN28 on OSCC tumorigenesis.

## 4 DISCUSSION

Emerging evidences have illustrated the important roles of cancer stem‐like cells (CSC) in human tumours, including OSCC.^12^, ^13^ In present study, our team aims to investigate the biological role of SNHG20 on the stemness and tumorigenesis of OSCC.

In the oncogenesis of OSCC, it has been verified that long non‐coding RNAs (lncRNAs) participate in a series of pathophysiological process.^14^, ^15^, ^16^ LncRNA SNHG20 is remarkably up‐regulated in multiple tumour tissues compared with adjacent non‐tumour tissues, predicting poor prognosis for patients, including hepatocellular carcinoma, colorectal cancer and so on.^17^, ^18^ In this research, our results found that SNHG20 was up‐regulated in OSCC tissue compared with adjacent normal tissue. Besides, SNHG20 was enriched in OSCC sphere cells compared with parental cells. Therefore, our data illustrate the poor indicator of SNHG20 for OSCC patients; moreover, it is closely correlated with OSCC cancer stem‐like properties.

The cancer stem‐like properties always make the tumorigenesis complex and cause the recurrence of residual tumour tissue.^19^, ^20^ Therefore, in consideration of the enrichment of SNHG20 in OSCC sphere, we suppose that SNHG20 regulates the stemness of OSCC to modulate its oncogenesis. Loss‐of‐function experiments indicated that SNHG20 knockdown inhibited the mammosphere‐forming, ALDH1 expression, stem factors (LIN28, Nanog, Oct4, SOX2) levels, suggesting the CSC‐inhibition of SNHG20 knockdown. Meanwhile, SNHG20 knockdown inhibited the proliferation and tumour growth of OSCC cells. Thus, our data and results conclude that the underlying mechanism of SNHG20 towards OSCC is the CSC properties regulation. The important function of lncRNA in human cancers has been verified. For example, lncRNA HOTAIR was upregulated in OSCC tumour tissues and OSCC cell lines; moreover, overexpression of HOTAIR enhanced the metastatic potential and epithelial‐mesenchymal transition (EMT) characteristics of OSCC.^21^ LncRNA CEBPA‐AS1 is up‐regulated in OSCC and functions as a potential oncogene, being correlated with poor differentiation, lymph node metastasis and high clinical stage via a novel pathway CEBPA/Bcl2.^22^

Although our results reveal the epigenetics role of SNHG20 on OSCC tumorigenesis and stemness, the specific biological functions should be carried out by functional proteins, instead of non‐coding RNA.^23^, ^24^ Therefore, we continue to investigate the underlying mechanism within SNHG20 and OSCC stemness. Bioinformatics tools and luciferase reporter assay reveal and validate that miR‐197 functions as a “linker” to connect SNHG20 and LIN28 by complementary binding at 3′‐UTR. Validation experiments confirm the associated function of SNHG20/miR‐197/LIN28 on OSCC tumorigenesis. The regulatory mechanism is described as competing endogenous RNA (ceRNA) theory.^25^, ^26^, ^27^, ^28^ LncRNAs harbour miRNAs and absorb dissociative miRNAs, decreasing the abundance in cells.^29^, ^30^ For instance, lncRNA NR2F1‐AS1 promoted ABCC1 expression through endogenous sponging miR‐363 to regulate hepatocellular carcinoma HCC oxaliplatin‐resistance.^31^ In this study, we found that SNHG20 functioned as a “sponge” for miR‐197, meanwhile, miR‐197 also targeted LIN28, forming a whole regulatory pathway. The ceRNA mechanism is a canonical theory for lncRNA in tumour tumorigenesis. For instance, lncRNA PDIA3P is overexpressed in OSCC and indicates the poor survival rate of OSCC patients, which negatively regulates miR‐185‐5p by targeting cyclin D2.^32^

In summary, our experiments preliminarily investigate the role of lncRNA SNHG20 on the OSCC tumorigenesis. Results reveal the important function of SNHG20/miR‐197/LIN28 axis in the oncogenesis and stemness of OSCC, suggesting the vital role of SNHG20 in OSCC.

## CONFLICT OF INTEREST

All authors declare no conflicts of interest.
