# Supplementary figures and images for: Long non‐coding RNA SNHG20 promotes the tumorigenesis of oral squamous cell carcinoma via targeting miR‐197/LIN28 axis
Source: J Cell Mol Med. 2018 Nov 5;23(1):680–8. doi: 10.1111/jcmm.13987 (PMC6307847; doi:10.1111/jcmm.13987)

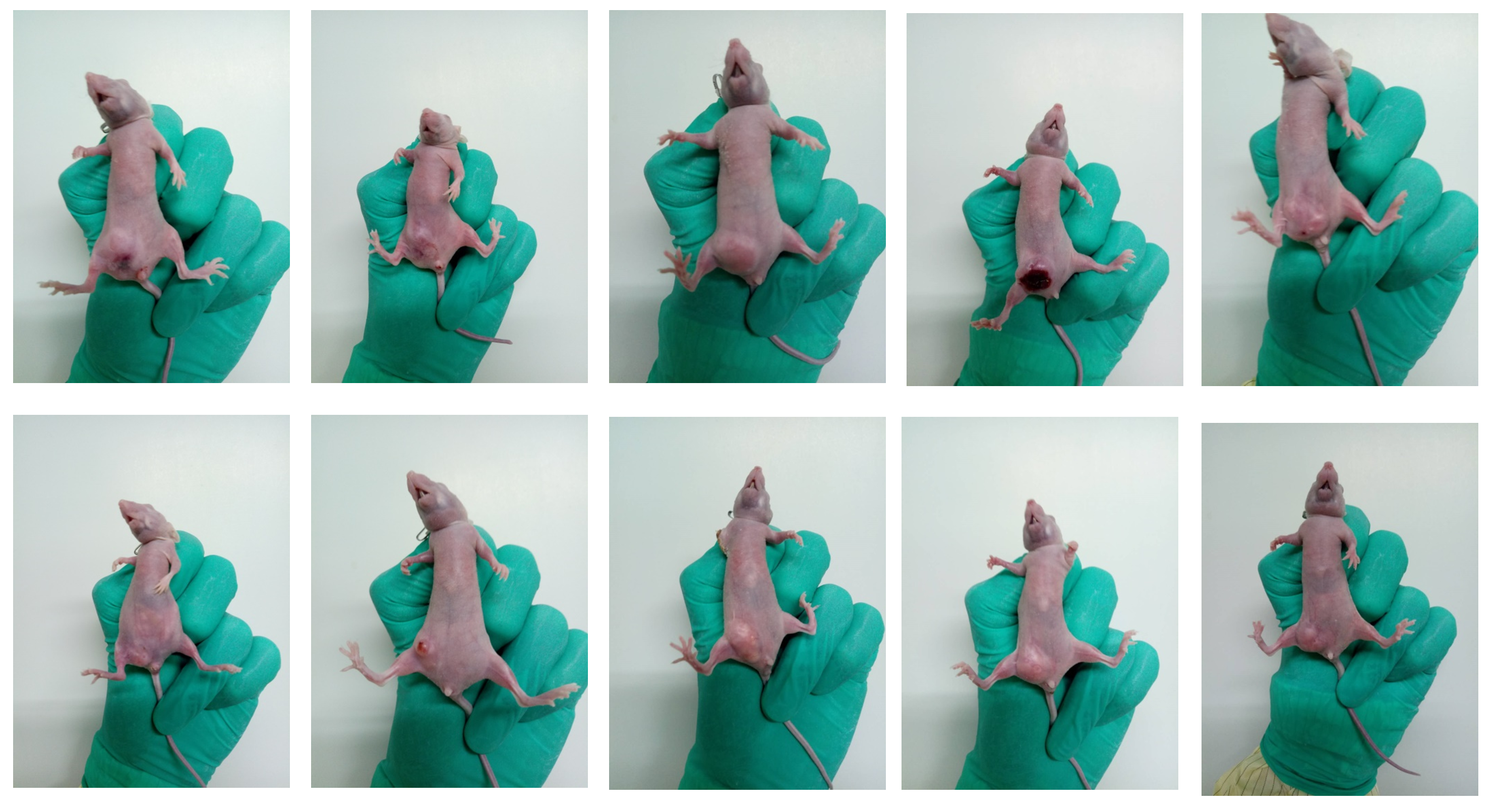

Supplement: Supplementary file 1 [file JCMM-23-680-s001.tif]

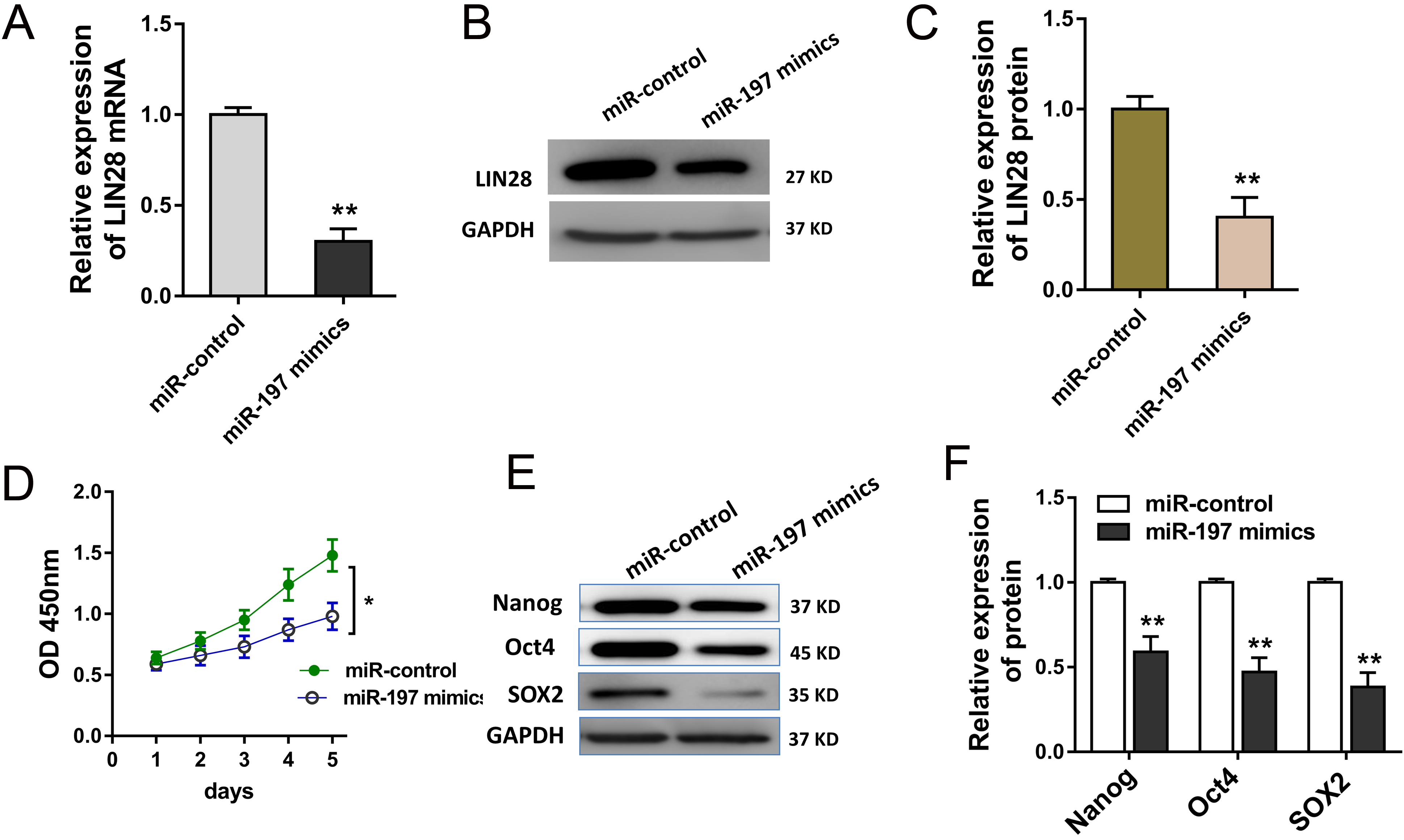

Supplement: Supplementary file 2 [file JCMM-23-680-s002.tif]

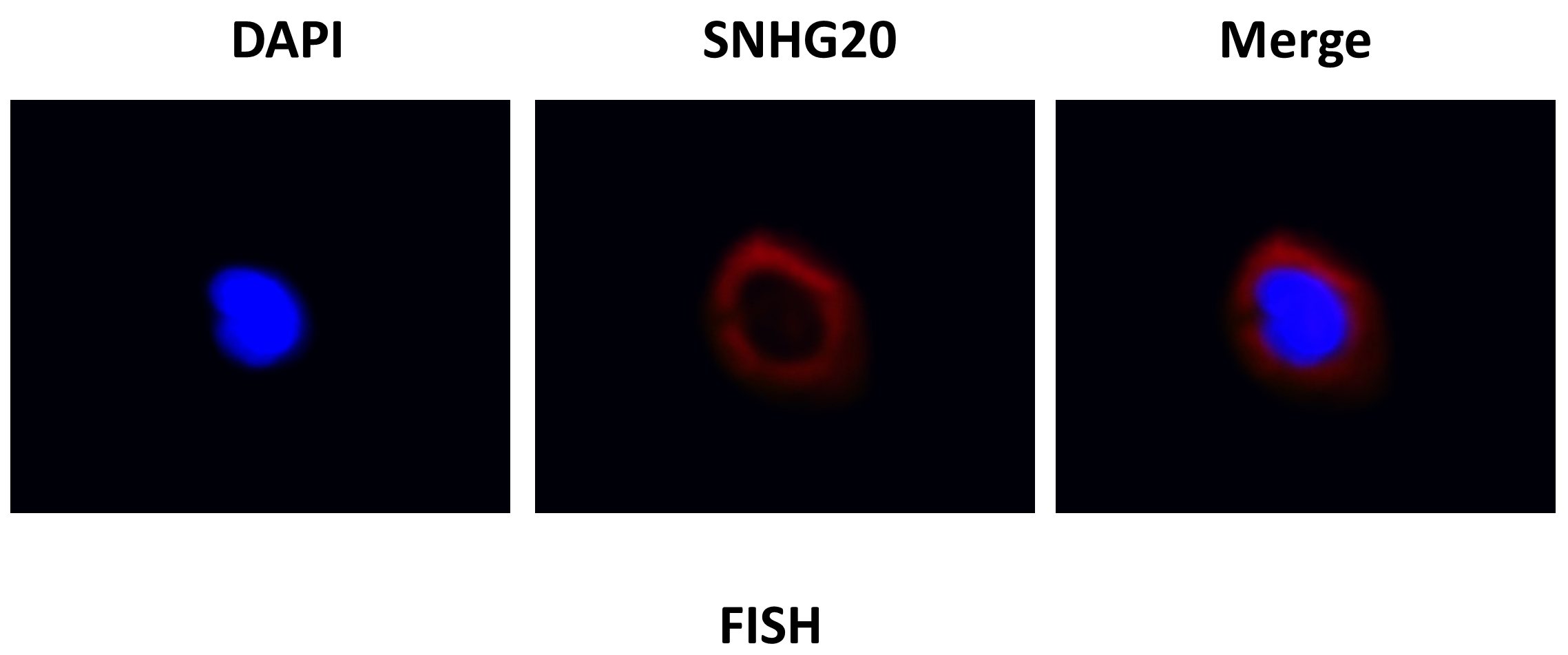

Supplement: Supplementary file 3 [file JCMM-23-680-s003.tif]
